# Predictive Model for Early Neurological Deterioration in Acute Ischemic Stroke Utilizing Novel Thrombotic Biomarkers

**DOI:** 10.1002/brb3.70577

**Published:** 2025-05-26

**Authors:** Yifei Zhao, Hao Zhu, Changfei Dai, Wen Liu, Wenjin Yu, Bin Yan, Xiyang Ji, Lin Li, Dong Wei, Zhaopan Li, Ping Chen

**Affiliations:** ^1^ Department of Neurology Xianyang Hospital of Yan’ an University Xianyang China; ^2^ Department of Neurology The First People's Hospital of Xianyang Xianyang China; ^3^ Department of Neurological Intensive Care Unit Xianyang Hospital of Yan'an University Xianyang China; ^4^ Institute of Brain Disease Xianyang Hospital of Yan'an University Xianyang China

**Keywords:** early neurological deterioration, plasmin‐α2 plasmin inhibitor complex, thrombin‐antithrombin complex, thrombomodulin, tissue prothrombin activator‐prothrombin activator inhibitor complex

## Abstract

**Background:**

Novel thrombotic molecular markers are significantly linked to acute ischemic stroke (AIS). However, the relationship between thrombin‐antithrombin complex (TAT), tissue plasminogen activator–inhibitor complex (t‐PAIC), plasmin‐α2 plasmin inhibitor complex (PIC), thrombomodulin (TM), and early neurological deterioration (END) remains unclear. Therefore, we developed a prediction model for END based on these markers and evaluated its accuracy and clinical utility.

**Methods:**

Retrospective analysis of patients diagnosed with AIS in our hospital from 2023–2024. The above patients were divided into a training set (N = 577) and a test set (N = 246) in a 7:3 ratio. Least absolute shrinkage and selection of operator regression (LASSO) valid predictors were used. The coefficients of the predictors in logistic regression were used to develop a nomogram and to validate its differentiation, calibration, and clinical utility.

**Results:**

The prevalence of END in AIS patients was 24.3%. Predictors screened according to LASSO regression analysis included age, the National Institutes of Health Stroke Scale (NIHSS) score, t‐PAIC, PIC, lymphocyte, and platelet. The resulting nomograms had the area under the curve (AUC) of 0.867 (95% CI, 0.834‐0.9) and 0.825 (95% CI, 0.757‐0.892) in the training and test sets, respectively, which had good differentiation. In addition, the calibration curve and decision curve analysis (DCA) showed that the model had good calibration and clinical utility.

**Conclusion:**

A predictive model for END was developed using the serological markers t‐PAIC (male >17.13 ng/mL;female >10.52 ng/mL), PIC >0.85 µg/mL, Lymph ≤ 3.2×10^9/L, NIHSS, age, and platelet. The model has significant predictive value for END occurrence in patients with AIS.

## Graphical abstract

This study developed a predictive nomogram for early neurological deterioration (END) in acute ischemic stroke (AIS) using serum biomarkers tissue plasminogen activator–inhibitor complex (t‐PAIC) and plasmin ‐α2 plasmin inhibitor complex (PIC), age, National Institutes of Health Stroke Scale (NIHSS) score, lymphocyte (lymph), and platelet (PLT). The model demonstrated strong predictive accuracy with well‐calibrated and clinical utility for END risk assessment.

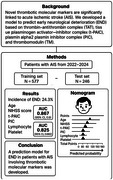

## 1 Introduction

Stroke stands as a leading cause of disability and mortality worldwide, contributing to approximately 1.6 million deaths annually in China alone (Tu et al. 2017). A significant portion of acute ischemic stroke (AIS) patients experience early neurological deterioration (END) within hours or days of onset (Boulenoir et al. 2021). Despite recent therapeutic advancements, END in AIS remains associated with serious clinical outcomes. The incidence of END among AIS patients varies from 5% to 40%, depending on the inclusion criteria and assessment methods used in studies (Hou et al. 2020; Kanamaru et al. 2017; Lee et al. 2017). Previous research indicates that patients experiencing END have a poor prognosis at 3 months and a high mortality rate (Heitsch et al. 2021; Liu et al. 2020; Thiebaut et al. 2018). Hence, it's crucial to identify the risk factors associated with END. However, the predictive capacity of individual factors is limited, necessitating the development of further prediction models.

END occurs by multiple and complex mechanisms, such as intracerebral hemorrhage, malignant edema, early recurrence of stroke, and hemodynamic problems such as poor collateral circulation, seizures, infections, and metabolism (Huang, Tsai, Lee, Yang, and Pan 2018; Seners, Turc, Oppenheim, and Baron 2015; Tanaka et al. 2020). Research indicates that acute ischemic stroke triggers changes in blood flow and disrupts the coagulation fibrinolytic system, leading to platelet activation, adhesion, and aggregation, culminating in blood clot formation and subsequent vessel narrowing or blockage (Liu et al. 2021; Seners et al. 2017). Several studies have underscored the significant association between novel thrombotic molecular markers, including thrombin‐antithrombin complex (TAT), tissue plasminogen activator–inhibitor complex (t‐PAIC), plasmin‐α2 plasmin inhibitor complex (PIC) and thrombomodulin (TM), with exacerbating thrombus formation, abnormal hypercoagulation, and impaired fibrinolysis in AIS patients. The relationship between these four indicators and END occurrence in AIS patients remains unclear, underscoring the need for further investigation.

Our study aimed to investigate discrepancies in four serological indicators between END and non‐END (NEND) patients. Subsequently, we constructed a predictive model and assessed its clinical utility. Our goal is to help clinicians quickly and efficiently assess the likelihood of END in their patients, thereby aiding clinical decision‐making.

## 2 Methods

### 2.1 Patients

A retrospective analysis was conducted on patients with acute ischemic stroke at the Cerebrovascular Hospital of Xianyang Hospital of Yan'an University between November 2022 and December 2024. The Ethics Committee of Xianyang Hospital of Yan'an University approved the study (Ethical approval number: YDXY‐KY‐2022‐008). All patients signed an informed consent form at the time of admission.

Inclusion criteria: (1) Age > 18 years, (2) Patients admitted to the hospital within 24 h of disease onset, and (3) The diagnostic criterion for AIS was acute neurological deficit due to focal ischemia, supported by cranial computed tomography (CT) or magnetic resonance imaging (MRI) evidence of acute stroke (Mendelson and Prabhakaran 2021).

Exclusion criteria: (1) Patients undergoing intravenous thrombolysis, endovascular therapy, or bridging therapy in an emergency, (2) Patients unable to cooperate with neurological function assessment due to impaired consciousness or serious comorbidities at admission, (3) Patients with a recent history of infection within the last 2 weeks, acute myocardial infarction, angina pectoris, venous thrombotic disease, or ischemic stroke within the last 3 months and (4) Use of antiplatelet drugs, anticoagulants, statins, etc., within the last month.

### 2.2 Data Collection

Upon admission, demographic data, including age and gender, were collected, and risk factors such as hypertension, diabetes, coronary heart disease, hyperlipidemia, smoking, and alcohol consumption were assessed. Two experienced neurologists evaluated stroke severity using the National Institutes of Health Stroke Scale (NIHSS) score. This assessment was repeated at 24 h, 3 days, and 7 days post‐admission, as well as whenever there was a change in the patient's condition. In the event of disagreement between the neurologists regarding NIHSS scores, a third neurologist was consulted to make the final assessment. Diagnostic criteria for END in stroke were defined as an increase in the NIHSS motor score by ≥1 point or total score by ≥ 2 points within 7 days of admission (Li et al. 2020). All patients mentioned above received antiplatelet therapy upon admission.

### 2.3 Biomarker Measurements

Blood samples were obtained from acute stroke patients within 24 h of their admission. The thrombotic markers TAT, t‐PAIC, PIC, and TM were determined using a sandwich immunoassay, which measures the levels of thrombotic markers in two steps. First, the samples, immunomagnetic beads coated with antibodies to TAT, t‐PAIC, PIC, and TM, are thoroughly mixed, incubated, and washed. In the second step, the corresponding antibody labeled with alkaline phosphatase is added to the cuvette and incubated to form a double antibody sandwich immune complex; at the end of the reaction, the complex is washed, and the luminescence intensity is measured by adding the substrate solution. Finally, the analyzer automatically calculates the result according to the stored calibration curve. Thrombus markers TAT, t‐PAIC, PIC, and TM were determined by aShine i2900 automated chemiluminescence immunoassay analyzer provided by Guangzhou Wondfo Biotech Co., Ltd. All tests were performed according to the manufacturer's instructions. The testing platform was not changed for all blood samples during this study.

### 2.4 Statistical Analysis

Patients with AIS were categorized into two groups based on the diagnostic criteria of END: the END group and the NEND group. Normally distributed measures are presented as mean ± standard deviation $\left(\bar{x}\pm S\right)$, while non‐normally distributed measures are expressed as median and quartile. Count data are presented as case numbers or percentages (*%*). Demographic characteristics, risk factors, and thrombotic biomarkers of the two groups were compared using the t‐test, Mann–Whitney *U* test, or chi‐square test to identify variables with predictive value. The dataset was randomly split into training and test sets in a 7:3 ratio for internal validation.

Predictor variable selection was conducted using the least absolute shrinkage and selection of operator regression (LASSO) regression technique, followed by multivariate logistic regression to construct the predictive model. The area under the curve (AUC) of the subjects' work characteristics and the consistency index (C‐index) were used to evaluate the discrimination of the predictive model.

Model calibration was evaluated using the Hosmer–Lemeshow test and calibration curves, while clinical utility was assessed through decision curve analysis (DCA). Statistical analyses were conducted using the Statistical Package for Social Sciences (SPSS) (version 25.0) and R software (version 4.3.2), with a significance level set at *p < 0.05*.

## 3 Results

### 3.1 Characteristics of Baseline Data

**FIGURE 1:**
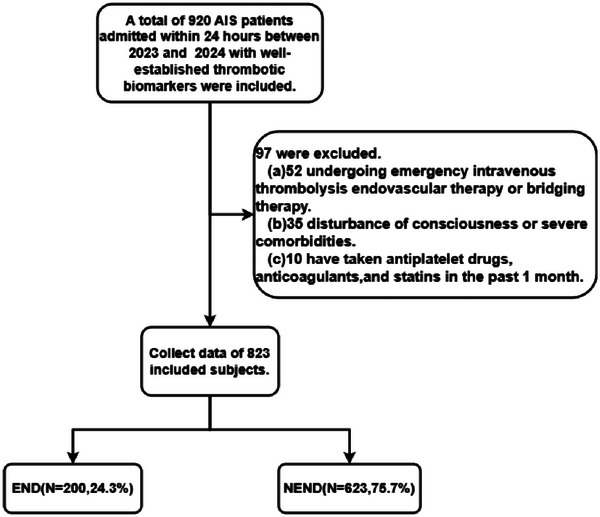
**Flowchart of patient selection. Abbreviations**: AIS, acute ischemic stroke; END group, an increase in the NIHSS motor score of ≥ 1 or a total score of ≥ 2 within 7 days of admission; NEND group, no increase in the NIHSS motor score or total score.

This study initially collected data from 920 patients with AIS. Of these, 52 patients received intravenous thrombolysis, endovascular therapy, or bridging therapy in the emergency department. Additionally, 35 patients were unable to undergo neurological assessment due to impaired consciousness or severe comorbidities, and 10 patients had recently taken antiplatelet drugs, anticoagulants, and statins within the past month. These patients were consequently excluded from the study, leaving a final cohort of 823 AIS patients for analysis. (Figure 1)

The mean age of the included patients was 64.58 ± 11.68 years, with 545 (66%) being male. Among the total admissions, 200 patients (24.3%) experienced END within 7 days of admission. There was no significant difference between the END and NEND groups in terms of gender, hypertension, diabetes mellitus, hyperlipidemia, history of smoking, history of alcohol consumption, systolic blood pressure (SBP), neutrophil, neutrophil‐to‐lymphocyte ratio (NLR), and platelet‐to‐neutrophil ratio (PNR) (*p > 0.05*). Compared to the NEND group, patients in the END group were older (mean age: 69.53 ± 11.41 years), had a higher prevalence of atrial fibrillation (AF) and higher baseline NIHSS scores, and there were more patients identified as having large‐artery atherosclerosis (LAA) and cardio embolic (CE) by Trial of ORG 10172 in Acute Stroke Treatment (TOAST) (*p* < 0.001). Among the coagulation indices, the proportions of TAT > 4.08 ng/mL, t‐PAIC (male > 17.13 ng/mL; female > 10.52 ng/mL), PIC > 0.85 µg/mL, and TM > 13.35 TU/mL in patients of the END group were 100%, 53%, 81%, and 14%, respectively, which were higher than those in the NEND group (*p <* 0.05). The PLT level of patients in the END group was 213.52 × 10^9/L, while it was 199×10^9/L in the NEND group, and the difference was statistically significant (*p <* 0.05). Among other serological indices, the proportion of WBC > 9.5 × 10^9/L, the proportion of lymphocyte count ≤ 3.2×10^9/L and PLR were higher in the END group than in the patients in the NEND group (*p < 0.05*). The proportion of neutrophil count > 6.3×10^9/L and NLR and PNR were also higher in the END group than in the NEND group, but the differences were not significantly different. (Table 1) The dataset was randomly divided into training and test sets at a 7:3 ratio for internal validation. Following a one‐way analysis of demographic characteristics, risk factors, baseline NIHSS score, SBP, TOAST, and serum markers in both the training and test sets, no significant differences were observed among the factors (*p >* 0.05). (Table 2) Common symptoms indicating neurological deterioration in END include gaze (4, 2.0%), visual field changes (3, 1.5%), facial movement impairment (12, 6.0%), motor dysfunction in the upper limbs (165, 82.5%), motor dysfunction in the lower limbs (148, 74.0%), limb ataxia (5, 2.5%), sensory deficits (14, 7.1%), language impairments (46, 23%), articulation difficulties (5, 2.5%), and extinction (3, 1.5%). (Table 3)

**TABLE 1:** Comparison of baseline data and thrombotic biomarkers between NEND and END groups.

Variables	NEND group (n = 623)	END group (n =200)	*p*‐value	t/χ2/U
Age	62.99 ± 11.32	69.53 ± 11.41	< 0.001^*^	−7.057
Gender (Male%)	423 (68)	122 (61)	0.088	2.919
Hypertension (%)	367 (59)	124 (62)	0.489	0.48
Diabetes mellitus (%)	117 (19)	35 (18)	0.763	0.091
Hyperlipidemia (%)	117 (19)	36 (18)	0.887	0.02
Atrial fibrillation (%)	112 (18)	62 (31)	< 0.001^*^	14.628
Cigarette smoking (%)	212 (34)	60 (30)	0.333	0.936
Alcoholism (%)	31 (5)	18 (9)	0.055	3.689
Baseline NIHSS	4 (2, 7)	9(5, 18)	< 0.001^*^	32052
SBP	148.69 ± 27.72	150.32 ± 26.81	0.46	−0.74
TOAST, (n%)			< 0.001^*^	42.564
Small‐artery occlusion	318 (51)	53 (26)		
Large‐artery atherosclerosis	218 (35)	90 (45)		
Cardio embolic stroke	87 (14)	57 (28)		
TAT>4.08 ng/mL, (%)	527 (85)	200 (100)	< 0.001^*^	33.409
PIC>0.85 µg/mL,(%)	255 (41)	162 (81)	< 0.001^*^	95.65
t‐PAIC(male>17.13 ng/mL;female >10.52 ng/mL),(%)	106 (17)	106 (53)	< 0.001^*^	100.644
TM >13.35 TU/mL, (%)	19 (3)	29 (14)	< 0.001^*^	34.087
WBC >9.5 × 10^9/L, (%)	0 (0)	40 (20)	< 0.001^*^	126.676
Neut >6.3 × 10^9/L, (%)	59 (30)	208 (33)	0.35	0.874
Lymph ≤ 3.2 × 10^9/L,(%)	21 (3)	29 (14)	< 0.001^*^	30.941
PLT	199 (182, 211.25)	213.52 (199.6, 229.05)	< 0.001^*^	83671
NLR	2.63 (1.71, 4.52)	3.13 (1.99, 4.55)	0.083	67369
PNR	37.65 (29.4, 55.69)	40.37 (31.21, 57.03)	0.061	67772
PLR	112.28 (72.92, 182.45)	125.61 (94.47, 185.12)	0.001^*^	71758

**Abbreviations**: END group, an increase in the NIHSS motor score of ≥ 1 or a total score of ≥ 2 within 7 days of admission; Lymph, lymphocyte; NEND group: no increase in the NIHSS motor score or total score; Neut, neutrophil; NLR, neutrophil‐to‐lymphocyte ratio; NIHSS: the National Institutes of Health Stroke Scale; PIC, plasmin alpha2 plasmin inhibitor complex; PLR, Platelet‐to‐Lymphocyte ratio; PLT, platelet; PNR, platelet‐to‐neutrophil ratio; SBP, systolic blood pressure; TAT, thrombin‐antithrombin complex; t‐PAIC, tissue plasminogen activator–inhibitor complex; TM, thrombomodulin; TOAST, Trial of ORG 10172 in Acute Stroke Treatment; WBC, white blood cell.

*p < 0.05 is considered statistically significant.

**TABLE 2:** Comparison of baseline data and thrombotic biomarkers between the training and test sets.

Variables	Test set (n = 246)	Training set (n = 577)	*p*‐value	t/χ2/U
END (%)	60 (24)	140 (24)	1	0
Age	64.19 ± 11.49	64.75 ± 11.76	0.525	−0.636
Gender (Male%)	167 (68)	378 (66)	0.563	0.335
Hypertension (%)	154 (63)	337 (58)	0.296	1.093
Diabetes mellitus (%)	47 (19)	105 (18)	0.834	0.044
Hyperlipidemia (%)	51 (21)	102 (18)	0.351	0.871
Atrial fibrillation (%)	63 (26)	111 (19)	0.05	3.827
Cigarette smoking (%)	69 (28)	203 (35)	0.056	3.65
Alcoholism (%)	19 (8)	30 (5)	0.215	1.538
Baseline NIHSS	5 (2, 8)	5 (2, 9)	0.918	71291
SBP	5 (2, 8)	5 (2, 9)	0.918	71291
TOAST, (n%)			0.974	0.052
Small‐artery occlusion	112 (46)	259 (45)		
Large‐artery atherosclerosis	92 (37)	216 (37)		
Cardio embolic stroke	42 (17)	102 (18)		
TAT >4.08 ng/mL, (%)	213 (87)	514 (89)	0.367	0.815
PIC >0.85 µg/mL,(%)	129 (52)	288 (50)	0.557	0.345
t‐PAIC(male >17.13 ng/mL;female>10.52 ng/mL),(%)	58 (24)	154 (27)	0.397	0.719
TM>13.35TU/mL, (%)	19 (8)	29 (5)	0.177	1.82
WBC >9.5 ×10^9/L, (%)	8 (3)	32 (6)	0.221	1.498
Neut >6.3 ×10^9/L, (%)	82 (33)	185 (32)	0.783	0.076
Lymph ≤ 3.2 ×10^9/L,(%)	15 (6)	35 (6)	1	0
PLT	208 (194.65, 225.96)	210.98 (196, 227.87)	0.376	68208
NLR	3.26 (1.98, 4.94)	2.88 (1.9, 4.48)	0.118	75857
PNR	38.35 (30.59, 55.41)	41.03 (31.12, 57.03)	0.384	68252
PLR	129.72 (92.29, 194.98)	120.73 (87.87, 180.26)	0.069	76649

**Abbreviations**: END, an increase in the NIHSS motor score of ≥ 1 or a total score of ≥2 within 7 days of admission; Neut, neutrophil; NLR, neutrophil‐to‐lymphocyte ratio; NIHSS: the National Institutes of Health Stroke Scale; PIC, plasmin alpha2 plasmin inhibitor complex; PLR, Platelet‐to‐Lymphocyte ratio; PLT, platelet; PNR, platelet‐to‐neutrophil ratio; SBP, systolic blood pressure; TAT, thrombin‐antithrombin complex; t‐PAIC, tissue plasminogen activator–inhibitor complex; TM, thrombomodulin; TOAST, Trial of ORG 10172 in Acute Stroke Treatment; WBC, white blood cell.

*p < 0.05 is considered statistically significant.

**TABLE 3:** Symptoms of neurological deterioration and number of patients in the END group.

	END group
Consciousness, n (%)	0(0)
Gaze, n (%)	4 (2)
Visual fields, n (%)	3 (1.5)
Facial movement, n (%)	12 (6.0)
Motor function (arm), n (%)	165 (82.5)
Motor function (leg), n (%)	148 (74.0)
Limb ataxia, n (%)	5 (2.5)
Sensory, n (%)	14 (7.0)
Language, n (%)	46 (23.0)
Articulation, n (%)	5(2.5)
Extinction or inattention, n (%)	3 (1.5)

**Abbreviation**: END: an increase in the NIHSS motor score of ≥ 1 or a total score of ≥ 2 within 7 days of admission.

### 3.2 Determination of the Best Predictors of the Occurrence of END in Patients With AIS

**FIGURE 2:**
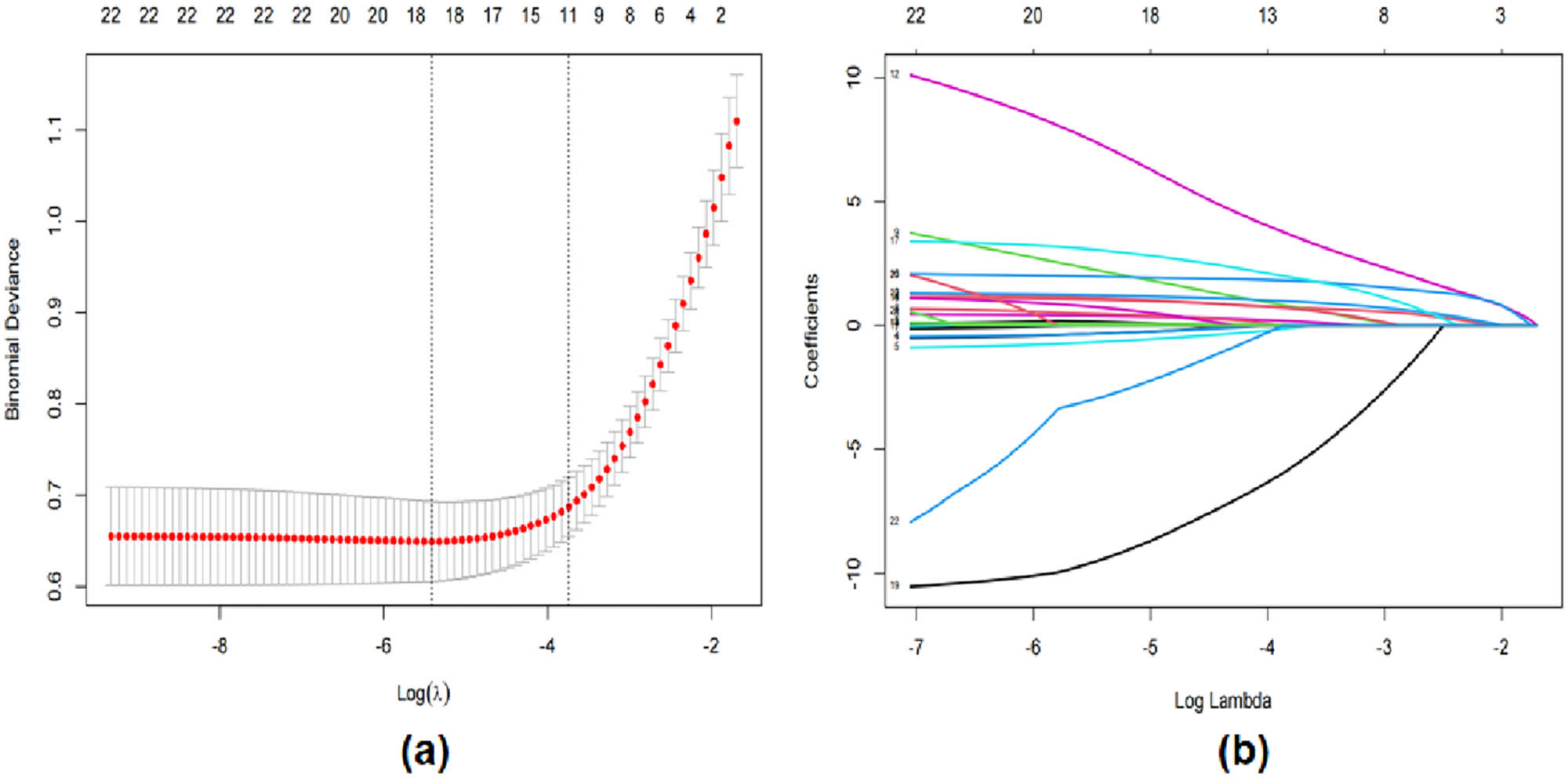
**Predictor selection using the LASSO regression analysis with tenfold cross‐validation. (a)** Tuning parameter (lambda) selection of deviance in the LASSO regression based on the minimum criteria (left dotted line) and the 1‐SE criteria (right dotted line). **(b)** A coefficient profile plot was created against the log (lambda) sequence. In the present study, the predictor's selection was according to the 1‐SE criteria (right dotted line), where 11 nonzero coefficients were selected. **Abbreviations**: LASSO, least absolute shrinkage and selection operator; SE, standard error.

From the 22 clinical variables collected above, LASSO regression analysis with 10‐fold cross‐validation was used, from which 11 variables with non‐zero coefficients were calculated, and these were age, hyperlipidaemia, AF, NIHSS, TOAST, t‐PAIC(male>17.13 ng/mL; female >10.52 ng/mL), TAT >4.08 ng/mL, PIC >0.85 µg/mL, WBC >9.5 × 10^9/L, Lymph ≤ 3.2 × 10^9/L, PLT. (Figure 2)

To further simplify the prediction model, the above 11 variables were subjected to multivariate logistic regression analysis, and finally, 6 variables were filtered out to be used in the generation of the column chart, including Age, NIHSS, t‐PAIC(male >17.13 ng/mL; female >10.52 ng/mL), PIC >0.85 µg/mL, Lymph ≤ 3.2 × 10^9/L, PLT. (Table 4)

**TABLE 4:** Multifactorial binary logistic regression analysis of the occurrence of END in patients with AIS.

	B	S.E.	Wald	OR	95%CI	*p*‐value
Age	0.043	0.011	15.691	1.044	1.022–1.067	<0.001^*^
Lymph ≤ 3.2 × 10^9/L	1.013	0.004	13.633	1.987	1.979–1.993	<0.001^*^
NIHSS Score	0.088	0.017	25.711	1.092	1.056–1.131	<0.001^*^
PLT	1.485	0.439	11.427	4.415	1.877–10.599	<0.001^*^
PIC >0.85 µg/mL	1.345	0.264	26.043	3.838	2.313–6.521	<0.001^*^
t‐PAIC(male >17.13 ng/mL;female >10.52 ng/mL)	0.935	0.256	13.302	2.548	1.537–4.209	<0.001^*^

**Abbreviations**: END, an increase in the NIHSS motor score of ≥ 1 or a total score of ≥ 2 within 7 days of admission; Lymph, lymphocyte; NIHSS, the National Institutes of Health Stroke Scale; PIC, plasmin alpha2 plasmin inhibitor complex; PLT, platelet; t‐PAIC, tissue plasminogen activator–inhibitor complex.

*p < 0.05 is considered statistically significant.

### 3.3 The Construction of Nomogram

**FIGURE 3:**
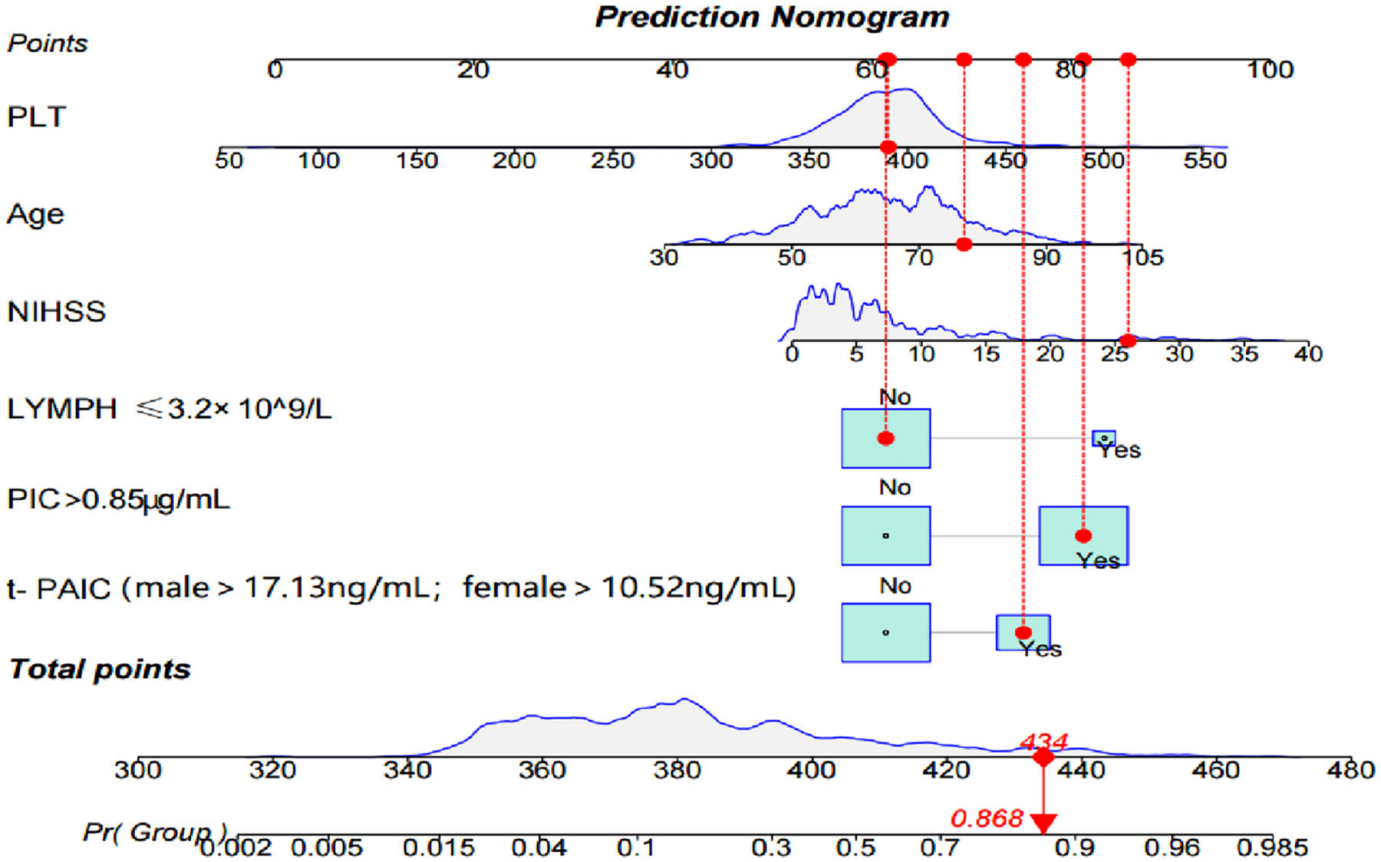
**Nomogram for predicting END risk and its algorithm**. First, a point was found for each variable of an END patient on the uppermost rule; then all scores were added together and the total number of points was collected. Finally, the corresponding predicted probability of END was found on the lowest rule. **Abbreviations**: END, an increase in the NIHSS motor score of ≥1 or a total score of ≥ 2 within 7 days of admission; Lymph, lymphocyte; NIHSS, the National Institutes of Health Stroke Scale; PLT, platelet; PIC, plasmin alpha2 plasmin inhibitor complex; t‐ PAIC, tissue plasminogen activator–inhibitor complex; 0, normal serum indicators; 1, abnormal elevation of serum markers.

Based on the six variables mentioned above, the R software was applied to generate a nomogram about the predicted risk of END occurrence. (Figure 3). The nomogram serves as a predictive tool for estimating the likelihood of a patient experiencing END. It assigns a certain number of points to each independent predictor of END. Each variable of the END patient receives a point value according to predefined rules, and these points are then summed to obtain a total score. This total score is used to determine the corresponding END prediction probability based on a predefined scale. An increase in the total nomogram score indicates a higher risk of END, while a decrease suggests a lower likelihood. For example, one of the patients was 77 years old and had the NIHSS score of 26 on admission, PIC > 0.85 µg/mL, t‐PAIC > 17.13 ng/mL, Lymph > 3.2×10^9/L, and a PLT count of 391 × 10^9/L. The patient's total score ended up being 434, and the probability of END was 0.868.

### 3.4 Internal Validation of the Nomogram

**FIGURE 4:**
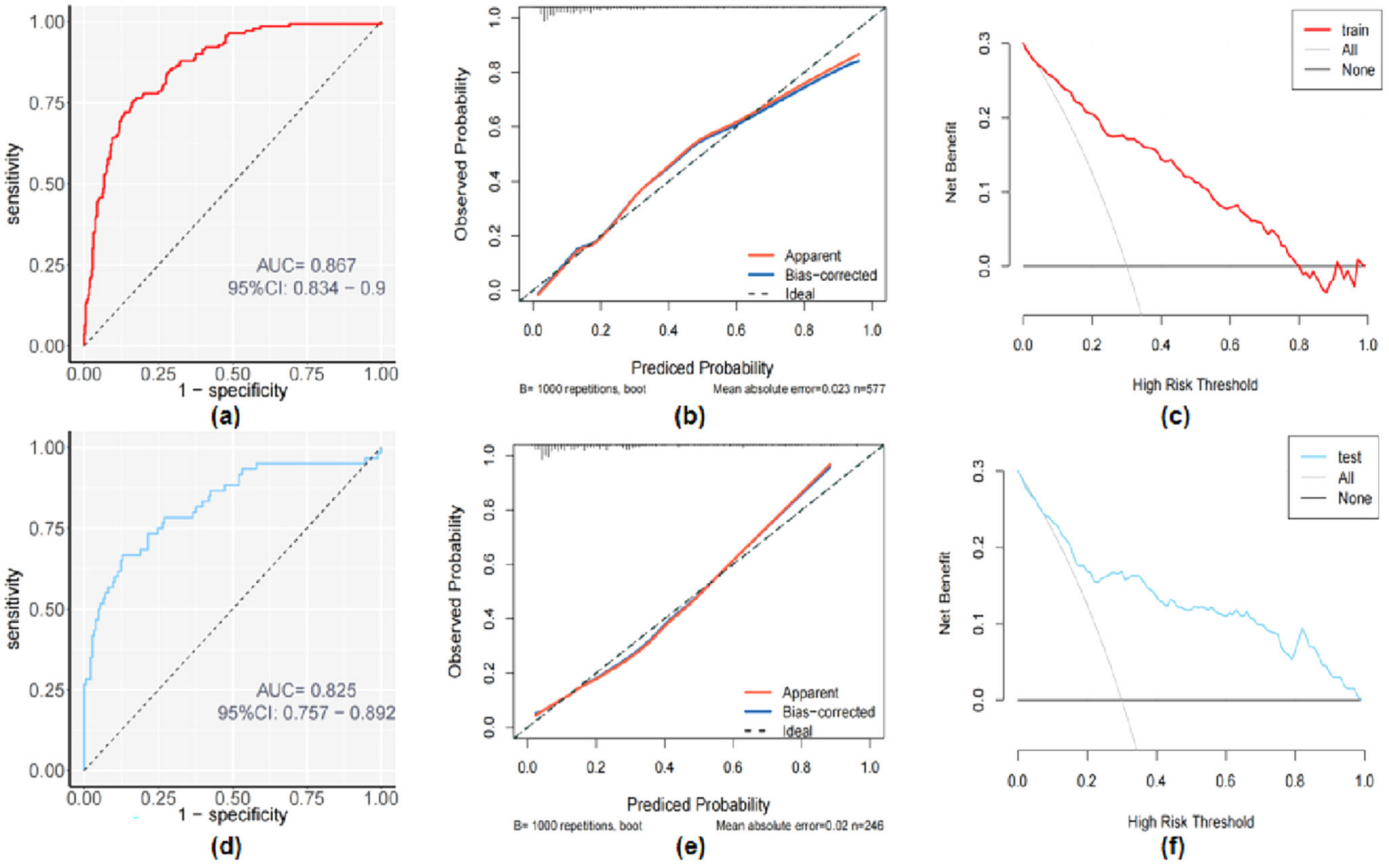
**Evaluation of the predictive model on train and test sets**.ROC curves **(a)**, calibration curves **(b)**, and DCA**(c)** for the nomogram in the training cohort. ROC curve **(d)**, calibration curve **(e)**, and DCA **(f)** for the nomogram in the test cohort. Calibration curve of the predictive model showing the degree of consistency between the predicted probability and observed probability. The figure includes a 45‐degree line that represents an ideal correction, indicating that the model's predictive power matches the actual risk of END perfectly. In the DCA, the X‐axis shows the probability of threshold; Y‐axis shows the net profit. And the Black line ensures that END will occur in all patients; Grey line ensures that all patients with AIS will not develop END.Red and blue line: AIS patients are predicted to progress to END. **Abbreviations**: AIS, acute ischemic stroke; DCA, decision curve analysis; END, early neurological deterioration; NIHSS, National Institutes of Health Stroke Scale; ROC, receiver operating characteristic.

The predictive model performed well, with an AUC of 0.867 (*95% CI, 0.834‐0.9*) for the nomogram among the 577 patients used as a training set, which exhibits remarkable accuracy and a good result from the Hosmer–Lemeshow test (*χ^2^ = 10.612, p = 0.225*), in addition to which the predicted probability of END was highly correlated with the actual probability, further demonstrating that the predictive model was well‐calibrated. Further plotting of DCA curves suggested that in the range of END probability thresholds from 0.05 to 0.8, compared with “all (assuming END occurs in all patients)” and “none (assuming END doesn't occur in all patients),” the calculated based on the column line plot END occurrence probability is closer to the actual occurrence probability and has good clinical utility. (Figures 4‐a, b, c)

In the test cohort, the value of AUC was 0.825 (*95% CI, 0.757–0.892*), suggesting that the model has high accuracy. In addition, the calibration curve showed good agreement between observed and predicted values. DCA reflected the same findings as the training set. In the range of END probability thresholds from 0.1 to 1.0, the application of nomograms for the prediction of the probability of END occurrence was more clinically useful compared with “all” and “none”. (Figures 4‐d, e, f)

## 4 Discussion

We conducted a retrospective study of AIS patients admitted within 24 h in 2022–2024 without thrombolysis therapy and built a nomogram to predict the probability of END using clinical indicators and novel thrombotic biomarkers. Our study showed that the prediction model constructed with age, NIHSS, t‐PAIC, PIC, lymphocyte, and PLT was effective in predicting the probability of END and had good clinical utility. This nomogram may help clinicians in the early identification of patients at high risk of END, thus guiding clinical decision‐making and better patient management.

The NIHSS is often used clinically to assess the degree of functional impairment due to stroke and consists of 11 tests, including the level of consciousness, gaze, visual field, facial paralysis, upper and lower limb movement, speech, and voice formation (Kwah and Diong 2014). The higher the score, the more severe the patient's stroke, and it is positively correlated with the size of the stroke (Duan et al. 2020; Hou et al. 2020). Our study found that higher NIHSS scores on admission in patients with AIS predicted a significantly higher likelihood of END, which is consistent with previous findings (Yu et al. 2017). Therefore, standardized and rigorous assessment of NIHSS scores in admitted patients should be carried out, and improved care and early intervention in patients with moderate to severe stroke may help to reduce the incidence of END and improve the survival of such patients. Our study also found that abnormally elevated lymphocytes were more common in non‐END patients, whereas END patients had normal or low lymphocyte levels, and NLR values were higher in END patients. The inflammatory response is an important pathophysiological mechanism in the development and progression of ischemic stroke (Rust, Grönnert, and Schwab 2018; Shi et al. 2019; Shichita et al. 2017; Xie et al. 2021). Based on previous findings, post‐ischemic brain tissue activates an inflammatory response that leads to the adhesion and production of inflammatory factors by leukocytes, including neutrophils, lymphocytes, and microglia. Neutrophils, as a major source of matrix metalloproteinase‐9 (MMP‐9), can disrupt the blood‐brain barrier, leading to further deterioration of the brain infarct (Ouk et al. 2019; Walz and Cayabyab 2017). Lymphocytes, on the other hand, are known to protect the blood‐brain barrier and improve neurological function, and lower levels of lymphocytes may predict a poor prognosis for stroke (Miró‐Mur et al. 2016; Ren et al. 2017). NLR has also been found to predict the occurrence of worse outcomes in patients with AIS (Lux et al. 2020; Ren et al. 2017).

Previous studies have identified elevated levels of TAT as an independent risk factor for the severity of AIS (Ye, Liu, Wang, Xu, and Wu 2020) and closely associated with poor prognosis in AIS patients^8^. Similarly, patients with massive cerebral infarction (MCI) exhibited significantly higher levels of serum PIC compared to those with non‐massive cerebral infarction (Zhao et al. 2022). When AIS occurs, increased intracranial pressure and cerebral edema occur in the lesion area, leading to hypoperfusion and collateral circulation dysfunction (Bourcier et al. 2023), causing irreversible tissue damage and oxidative stress, stimulating coagulation abnormality, vascular wall damage, and permeability alteration (Kim et al. 2019), which in turn causes in situ thrombosis or re‐progression (Seners et al. 2017), leading to the occurrence of END and poor prognosis. AF‐related cardiogenic stroke is a known risk factor for END. Elevated levels of TAT and PAI‐1 are significantly associated with thromboembolic events and stroke in AF patients (Wu et al. 2015). Moreover, markers of coagulation and hemostatic activation (MOCHA) can predict AF following cryptogenic stroke (Ellis et al. 2018), as AF alters local blood flow, leading to coagulation activation, increased fibrinolytic biomarkers, and vascular endothelial damage. The thrombi observed in AIS and acute myocardial infarction exhibit differences in platelet and fibrin abundance, as well as the inflammatory cell count in each thrombus area (Koupenova et al. 2017; Shibata et al. 2015). Fibrin clots formed in AF are more compact than intracranial atherosclerotic plaques, leading to larger‐volume thrombi that block major intracranial vessels. This complexity exacerbates coagulation fibrinolytic system dysregulation, potentially contributing to END. PLT is an important component of the blood, and its activation secretes and expresses a variety of coagulation and inflammatory mediators that promote the formation of atherosclerotic plaques, which can lead to the development of AIS (Franco et al. 2015).

Various previous studies have been conducted to construct prediction models for END, and they included different populations, mainly patients who received endovascular thrombolysis and thrombolytic therapy, and the definition of early neurological deterioration in these studies was not identical. Lyu et al. investigated the incidence of END in patients after intravenous thrombolysis and concluded that NIHSS, SBP, and PLR were independent risk factors for END (Lyu et al. 2023). The Jin M team classified post‐thrombolytic END patients into symptomatic bleeding and non‐hemorrhagic factors, and the prediction model of non‐hemorrhagic END constructed with SBP, NIHSS, and LAO had a good discriminatory degree (*AUC = 0.811, 95% CI, 0.710–0.912*) (Jin et al. 2023). The logistic regression model constructed by Gong et al. concluded that NLR, PLR, and lymphocyte‐to‐monocyte ratio (LMR) were independent risk factors for END after thrombolysis (Gong et al. 2021). The above studies used NIHSS, SBP, and inflammatory factors as predictors of END, which are similar to the findings of the present study. XIE et al. combined NIHSS scores with imaging data and found that models constructed with higher NIHSS scores, middle cerebral artery stenosis, and carotid artery stenosis (> 50%) had a high predictive value for the occurrence of END (Xie et al. 2021). However, some critically ill patients are unable to cooperate with MRI in the early stages of the disease, making the practical application of the model difficult.

We used LASSO regression analysis to screen predictors and created a nomogram to assess the predictive value of age, NIHSS, t‐PAIC, PIC, lymphocyte, and PLT for END. Our predictors included serological indicators (coagulation, fibrinolysis markers, inflammatory factors), clinical severity (NIHSS score), and individual patient indicators (age), which predicted END by different mechanisms. And the above indicators are simple and easy to obtain, which helps clinicians in practical applications. Compared with univariate analyses, LASSO regression analyses have superior performance in screening predictors. We evaluated the discrimination and calibration of the prediction model by analyzing the receiver operating characteristic (ROC) and calibration curves. In addition, we used DCA, a statistical method, to compare models and determine their utility for clinical decision‐making (Van Calster et al. 2018). Our DCA results indicate that the model has high utility. Within the threshold range, the net benefit of the prediction model exceeded that of “all (assuming END occurs in all patients)” and “none (assuming END doesn't occur in all patients).” Therefore, the use of this prediction model to assess patients with AIS may help to identify the high‐risk group for END at an early stage, thus guiding clinicians in early intervention and related clinical decisions.

Despite the strengths of our study, some limitations should be acknowledged. First, this was a single‐center retrospective study, which may have introduced selection bias due to the small sample size. Our study only included patients with AIS who were not treated with thrombolytic therapy, and the model has not been used in clinical practice to assess the incidence of END and to evaluate the effect after early intervention. In addition, the prediction model was only internally validated and lacked external validation. In the future, we will include more multicenter samples, compare the differences in risk factors for END between thrombolytic and non‐thrombolytic patients, and evaluate the utility of the prediction model in guiding patient treatment in clinical practice. Despite these limitations, the clinical utility of the model is clear and provides a valuable tool for predicting END using simple serum marker tests.

## 5 Conclusion

A predictive model for END was developed using the serological markers t‐PAIC, PIC, lymphocyte, NIHSS score, age, and platelet. The model has significant predictive value for END occurrence in patients with AIS.

## Author Contributions

**Yifei Zhao**: writing–original draft, software, data curation, and visualization. **Hao Zhu**: methodology, writing–original draft, and validation. **Changfei Dai**: investigation and resources. **Wen Liu**: investigation and resources. **Wenjin Yu**: investigation and resources. **Bin Yan**: investigation and resources. **Xiyang Ji**: investigation and resources. **Lin Li**: investigation and resources. **Dong Wei**: investigation and resources. **Zhaopan Li**: investigation and resources. **Ping Chen**: conceptualization, supervision, formal analysis, project administration, writing–review and editing, validation, and funding acquisition.

## Ethics Statement

The Ethics Committee of Xianyang Hospital of Yan'an University approved the study (Ethical approval number: YDXY‐KY‐2022‐008.

## Consent

All authors reviewed the final version of the manuscript and approved it for publication. All patients signed an informed consent form at the time of admission.

## Conflicts of Interest

The authors declare no conflicts of interest.

### Peer Review

The peer review history for this article is available at https://publons.com/publon/10.1002/brb3.70577

## Data Availability

All data generated or analyzed during this study are included in this published article.
